# webTDat: A Web-Based, Real-Time, 3D Visualization Framework for Mesoscopic Whole-Brain Images

**DOI:** 10.3389/fninf.2020.542169

**Published:** 2021-01-13

**Authors:** Yuxin Li, Anan Li, Junhuai Li, Hongfang Zhou, Ting Cao, Huaijun Wang, Kan Wang

**Affiliations:** ^1^School of Computer Science and Engineering, Xi'an University of Technology, Xi'an, China; ^2^Shaanxi Key Laboratory of Network Computing and Security Technology, Xi'an, China; ^3^Wuhan National Laboratory for Optoelectronics, Britton Chance Center for Biomedical Photonics, Huazhong University of Science and Technology, Wuhan, China; ^4^MoE Key Laboratory for Biomedical Photonics, School of Engineering Sciences, Huazhong University of Science and Technology, Wuhan, China; ^5^HUST-Suzhou Institute for Brainsmatics, JITRI Institute for Brainsmatics, Suzhou, China

**Keywords:** visualization, 3D volume, terabyte images, software framework, whole-brain imaging

## Abstract

The popularity of mesoscopic whole-brain imaging techniques has increased dramatically, but these techniques generate teravoxel-sized volumetric image data. Visualizing or interacting with these massive data is both necessary and essential in the bioimage analysis pipeline; however, due to their size, researchers have difficulty using typical computers to process them. The existing solutions do not consider applying web visualization and three-dimensional (3D) volume rendering methods simultaneously to reduce the number of data copy operations and provide a better way to visualize 3D structures in bioimage data. Here, we propose webTDat, an open-source, web-based, real-time 3D visualization framework for mesoscopic-scale whole-brain imaging datasets. webTDat uses an advanced rendering visualization method designed with an innovative data storage format and parallel rendering algorithms. webTDat loads the primary information in the image first and then decides whether it needs to load the secondary information in the image. By performing validation on TB-scale whole-brain datasets, webTDat achieves real-time performance during web visualization. The webTDat framework also provides a rich interface for annotation, making it a useful tool for visualizing mesoscopic whole-brain imaging data.

## Introduction

The brain is one of the most complex organs in nature. Due to the large number of neurons in the brain and the complex structural connections between them, mapping a brain-wide network at mesoscopic resolution is critical for revealing brain function mechanisms (Lichtman and Denk, 2011; Koch and Reid, 2012). With the development of transgenic technology, neuronal tracers, and optical imaging, mesoscopic-scale whole-brain imaging has become a vital imaging technology for obtaining high-resolution brain connectivity information throughout the entire brain (Osten and Margrie, 2013; Mitra, 2014). These technologies include micro-optical sectioning tomography (MOST) (Li et al., 2010; Gong et al., 2016), serial two-photon tomography (STP) (Ragan et al., 2012; Economon et al., 2016), and light-sheet microscopy (LSM) (Niedworok et al., 2012), which can rapidly scan a complete mouse brain at submicrometric resolution in three dimensions or multiple dimensions. However, such scans comprise more than 10 TB of image data (Li et al., 2019), making them too large to be processed by typical computers. The large size of mesoscopic whole-brain data has posed a significant challenge to efficient visualization of and interaction with these datasets (Helmstaedter and Mitra, 2012), which are basic and essential tasks in the bioimage analysis pipeline (Peng, 2008; Walter et al., 2010; Meijering et al., 2016).

In recent years, many excellent large-scale image visualization methods have emerged in the field of bioimage informatics. The basic strategy for visualizing large-scale images is to use a multiresolution technique and image dicing. Such operations divide the data into many small blocks and store multiple copies of data at different resolutions. Subsequently, based on the resolution of the rendering window (screen), the image blocks with an appropriate resolution are loaded for visualization. According to the different data presentation methods, these methods can be classified into three categories: two-dimensional (2D) cross-sectional views, tri-planar (including arbitrary cross-sectional) views, and 3D visualizations. To construct 2D cross-sectional views, the early proposed methods mostly used 2D cross-sectional software such as CATMAID (Saalfeld et al., 2009) and Zoomify (Zoomify Inc, California, US). These methods process each 2D tomography image in a 3D image stack separately and then display them in 2D. These methods are similar to Image/Fiji, a widely used image analysis tool. By switching among different layers along one dimension, the complete 3D image can be browsed. Each two-dimensional tomography image is stored as a quadtree. Low-resolution images are displayed when viewing large-range images and high-resolution images are displayed when viewing small-range images, similar to Google Maps. The tri-planar view is an extension of the 2D cross-sectional view that displays all the XY, XZ, and ZY cross-sectional planes simultaneously. The typical methods include SSECRETT (Jeong et al., 2010), M-DIP (Lin et al., 2013), KNOSSOS (webKNOSSOS) (Helmstaedter et al., 2011; Boergens et al., 2017), BigDataViewer (Pietzsch et al., 2015), Neuroglancer (https://github.com/google/neuroglancer), etc. These methods select a region of interest (ROI) from the data and then visualize it in multiple 2D cross-section views (tri-view or arbitrary 2D cross-section view). However, these cross-sectional view-based methods are not intuitive enough to observe or understand the 3D information in the volumetric image (Long et al., 2012; Bria et al., 2016). Therefore, in recent years, various 3D-based massive image visualization methods have been proposed, including Terafly (Bria et al., 2016), Amira-XLVolume (FEI, Mérignac Cedex, France), Imaris-IMS (Oxford Instruments plc, Abingdon, UK) and our previous work, TDat (Li et al., 2017). These methods are based on specific image formats similar to an octree, allowing 3D ROIs to be selected directly from the massive data and rendered, providing a better visualization approach.

However, due to the massive data volumes, storing the data in local computers for processing, analysis, and visualization means that making many data copies so that researchers can work collaboratively difficult. Consequently, processing and visualizing the data stored on the server side through the network has become a developing trend in this field. With the development of 5G and cloud computing technology, which will be applied in the field of bioimage informatics, the need for web visualization of large-scale image data has become increasingly important and necessary. Because 3D visualization uses larger data than does 2D visualization, transferring and visualizing such high volumes of data via the Internet is time-consuming. This situation means that only a few 2D-based visualization methods can realistically be applied on the web. In fact, most of the above 3D-based visualization methods are limited to only local computers. Consequently, the existing solutions cannot solve the bottleneck imposed by the need for massive 3D volume transmissions over limited network bandwidth.

To address this problem, we developed webTDat, a web-based, real-time 3D interactive visualization framework for mesoscopic-scale whole-brain imaging datasets. webTDat adopts a fine-grained data storage format that separates images by pixel bit depth. This approach reduces the granularity during data accesses and reduces the total size of the data needed for visualization by up to 70% (half-bit mode). webTDat also includes a method to dynamically render image pixel bits that achieves an excellent response time (less than 400 ms); this approach preferentially renders the image's primary information and returns the semifinished image to users for interactive visualization. Simultaneously, the image is continuously refreshed in the background. webTDat includes a set of easy-to-handle interactive logical operations. For example, simple mouse drag-and-click operations can be used to accurately navigate through the massive data. The webTDat framework not only provides interactive visualization functions but also offers a rich interface for annotation. Moreover, webTDat can be applied to almost all types of mesoscopic whole-brain imaging data.

## Materials and Methods

### Architecture

webTDat uses a conventional client-server (C/S) architecture for data storage and retrieval. The raw image data are converted to a fine-grained hierarchical data format stored in many 3D files called bitBlocks. Each bitBlock contains only one bit-plane of the information of the corresponding block. Each dataset also includes metadata that describe other necessary information about the data, such as the dataset size, the original data resolution, file format, bit depth, level size, and file location. Both the image data and the metadata are stored on the server-side (the webTDat server). A client (webTDat viewer) first loads the metadata and then requests and visualizes the image data interactively. The server transmits bitBlocks to the client through the network protocol; the bitBlocks are merged into a volume of interest (VOI) for rendering and interactions on the client side.

### Bit-Plane Separated Fine-Grained Data Format

A multiresolution pyramid or octree is often used for large-scale volume data storage. The whole volume is subsampled recursively to create a hierarchy of resolutions. Each volume with a different resolution is split into fixed-size 3D blocks. When data are being accessed or visualized, only a small number of 3D blocks need to be read at a suitable resolution level. Based on this conventional hierarchical structure and our previous work on TDat, we designed a bit-plane (bit-level)-separated, fine-grained data format to enable more efficient data access. webTDat splits each 3D block into smaller files (bitBlocks) based on pixel bit depth.

**Figure 1:**
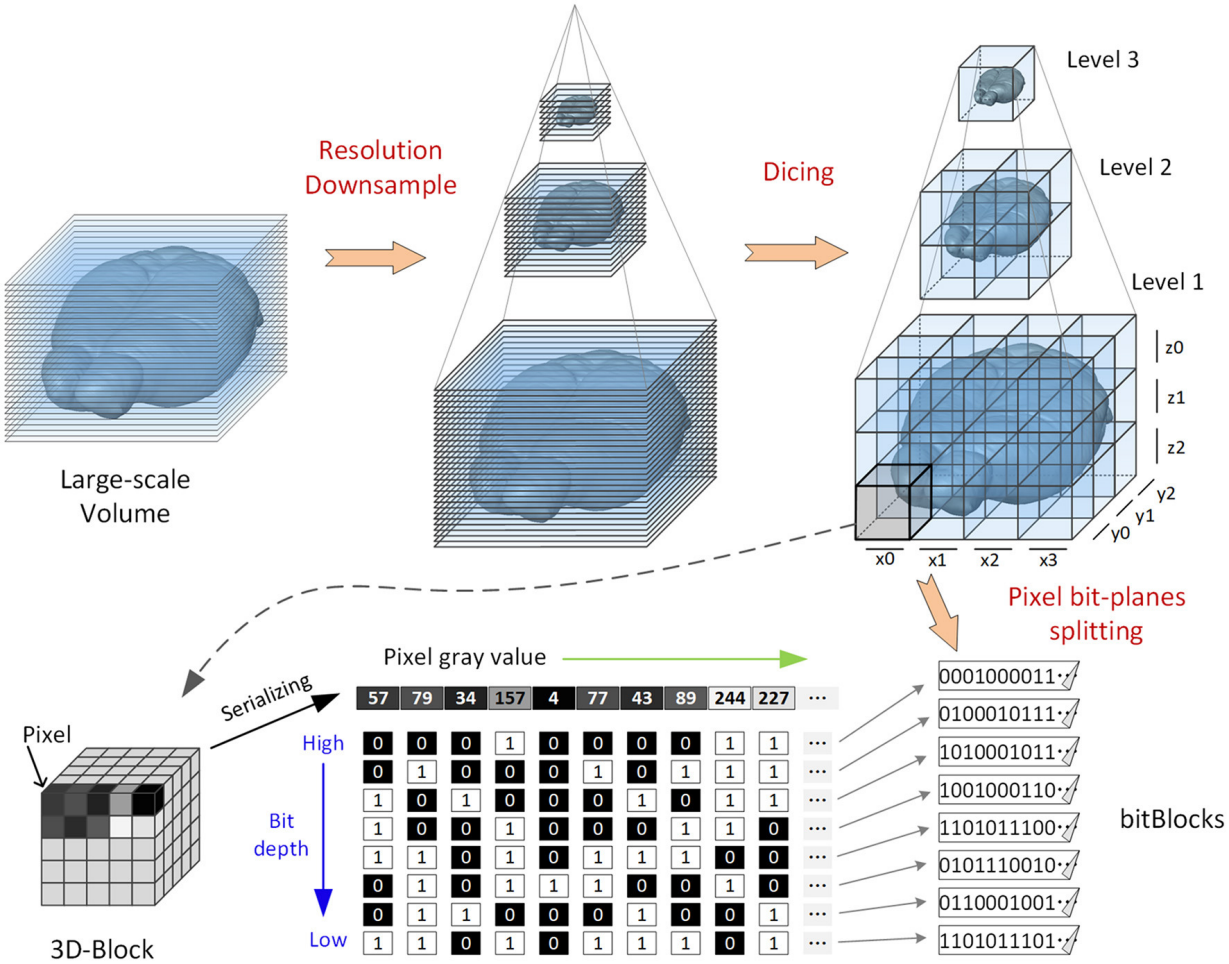
Illustration of a bit-plane-separated, fine-grained data format. The original volumetric data are recursively downsampled to generate three datasets with different resolution levels (for illustrative purposes). Each dataset is first diced into 3D blocks; then, the 3D blocks are split by bit depth, and the split results (bitBlocks) are stored separately.

The main data transformation processes are as follows (Figure 1): (1) Resolution downsampling: The raw volume is downsampled by 2 × recursively in the x, y, and z directions to obtain volumes with different resolutions; this process is conducted iteratively until the size of the lowest-resolution volume is <128(x) × 128(y) × 128(z). (2) Dicing: For all resolutions, each volume is split into nonoverlapping 3D blocks, each with a fixed size of 128 × 128 × 128 voxels. (3) Pixel bit-plane splitting: Each block is divided into different files according to pixel bit depth. The gray value of each pixel is split into multiple (eight or 16) binary values. The binary values on the same bit-plane belonging to different pixels are merged into a binary sequence. Each sequence contains the information of one block for a single bit-plane. Every eight bits in the sequence are stored into one byte (note that the values of these bytes have no actual meaning). LZW lossless compression is used to form the final encoded file (suffix of. *tif* ), which is called a bitBlock. This bit-plane separated storage method does not increase the data volume: it simply reorganizes the pixel value bits in each block into multiple files. Taking an 8-bit image volume as an example, each block is split into eight bitBlocks that store eight gray levels.

Thus, the information quantity from the highest bit to the lowest in the image is gradually reduced. Images split along bit-planes can be used to render high-bit information preferentially when visualized. The low-bit information is rendered later or not at all, which reduces the amount of data that need to be loaded to form an effective visualization.

**Figure 2:**
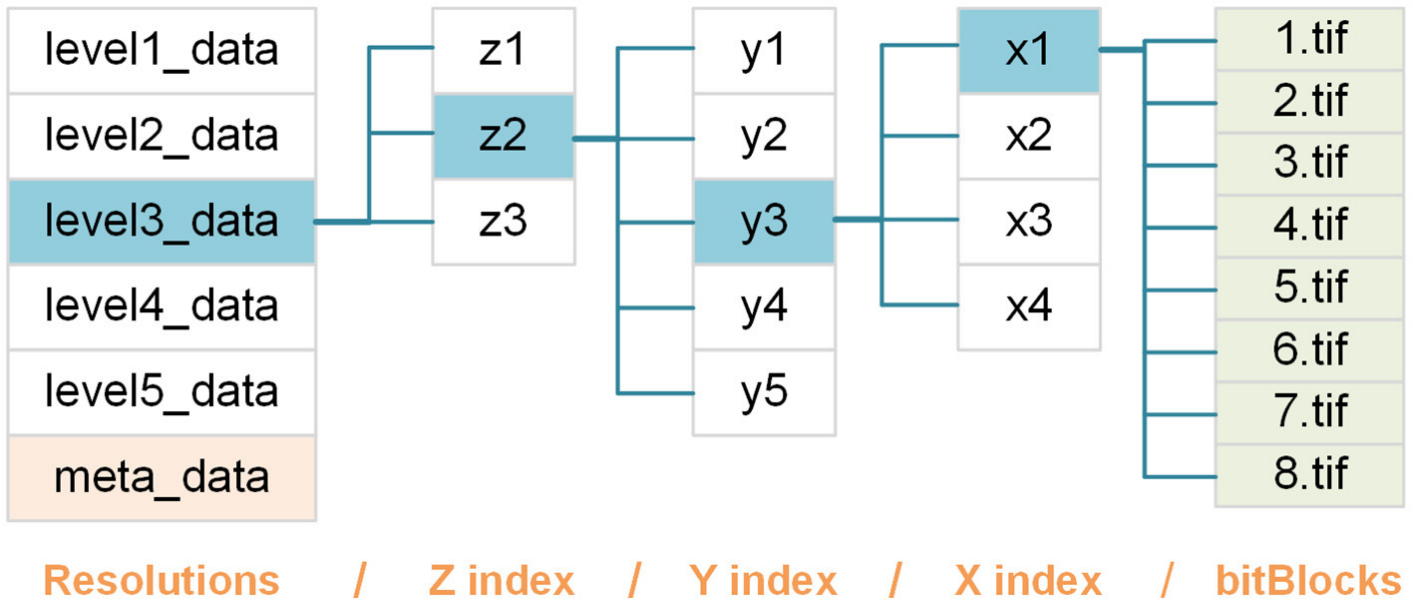
Schematic of the webTDat format. We take a set of whole mouse brain data that has been converted to webTDat format as an example. The bitBlocks are organized as a four-level hierarchy of folders according to their levels and locations in the data space.

A bitBlock file is named *b*.tif based on the bit-plane it contains; thus, *b* represents a specific bit-plane and is indexed from high to low. All bitBlocks are organized in a hierarchy of nested folders composed of four levels (Figure 2), defined as follows:

res: this folder level contains datasets with different resolutions, each of which is stored in a folder named level*r*_data, where *r* = 1 denotes the original resolution, and the resolution corresponding to *r* + 1 is a 2 × downsampling of the resolution corresponding to *r*.

z: this folder level contains bitBlocks with the same z(depth) position. Each group of bitBlocks is stored in a folder named z*i*, where *i* refers to the index of bitBlocks in the z-direction.

y: this folder level contains bitBlocks at the same y (height) position. Each group of bitBlocks is stored in a folder named y*j*, where *j* refers to the index of bitBlocks in the y direction.

x: this folder level contains bitBlocks with the same x (width) position. Each group of bitBlocks is stored in a folder named x*k*, where *k* refers to the index of bitBlocks in the x direction.

Using this hierarchical folder structure each bitBlock has a uniquely indexed address (URL).

A reformatting tool, webTDat reformatter, converts an original dataset into the webTDat data format. The webTDat reformatter adopts the same FPR data reformatting method as TDat but adds the functionality for generating bitBlocks to FPR (Li et al., 2017).

### Dynamic Rendering of Image Pixel Bits

**Figure 3:**
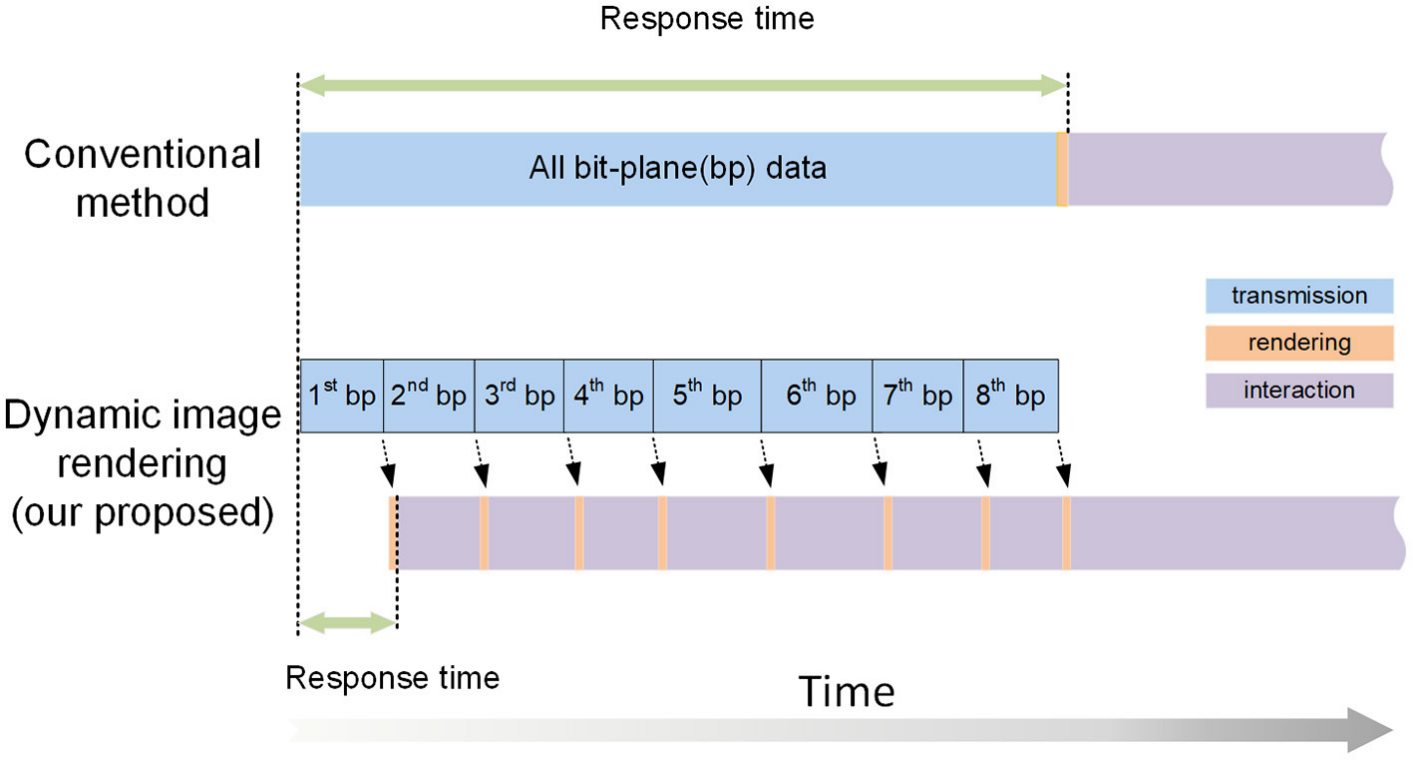
An illustration of dynamic image rendering. The squares of different colors represent the processes of data transmission, rendering, and interactive visualization. Our proposed method transmits the data for each bit-plane individually to achieve a short response time. In contrast, the conventional methods must transfer all the data before interactive visualization can begin.

Web visualization requires transmitting image data from the server to the client through the network. Compared with 2D data, transmitting 3D volume data requires more bandwidth and consumes more time, which increases the response time during visualization. Therefore, based on the bit-plane-separated, fine-grained data format, we propose a dynamic rendering of image pixel bits technology, which improves the response time of interactions and allows users to browse rapidly during web visualization. This method prioritizes transmitting the high bit-plane data. After a bit-plane of data has been transferred, the client renders the data immediately, displaying it for users for interaction and visualization. While interacting with the current data, the data for the other bit-planes are transferred synchronously to the client, which uses the new data to refresh the image dynamically (Figure 3).

The process works as follows: two threads on the client side are used to accomplish data transmission (*T* thread) and interactive rendering (*R* thread), respectively. The *T* thread downloads the bitBlocks needed for current visualization requests from the server. The high bit-plane bitBlocks are transferred first. When the bitBlocks of the first bit-plane have been completely transferred, the *T* thread sends a signal to the *R* thread, which decodes the transferred bitBlocks to generate a VOI containing only one bit-plane and renders it, allowing user interaction to begin as soon as possible. Then, even while the user is interacting with the current VOI, the *T* thread continues to requires and transfer the bitBlocks for the second bit-plane. After the second bit-plane transmission is completed, the *R* thread is notified by the T thread, and it combines these new bitBlocks with the current VOI, which is dynamically refreshed and now displays information for two bit-planes. Similarly, the bitBlocks of the other bit-planes are transferred and dynamically rendered during the VOI interaction. Because data rendering consumes little time, dynamic rendering does not have a significant impact on data interaction.

webTDat also provides an optional half-bit model that transfer only half of the bitBlocks (for example, for an 8-bit image, only the highest four bits of data are transmitted), which effectively reduce the time and bandwidth consumed by data transmission. When the image contrast is high, the image display shows no apparent differences between the half-bit and full-bit models.

### Volume Interactions Based on Drag-Click Operations

**Figure 4:**
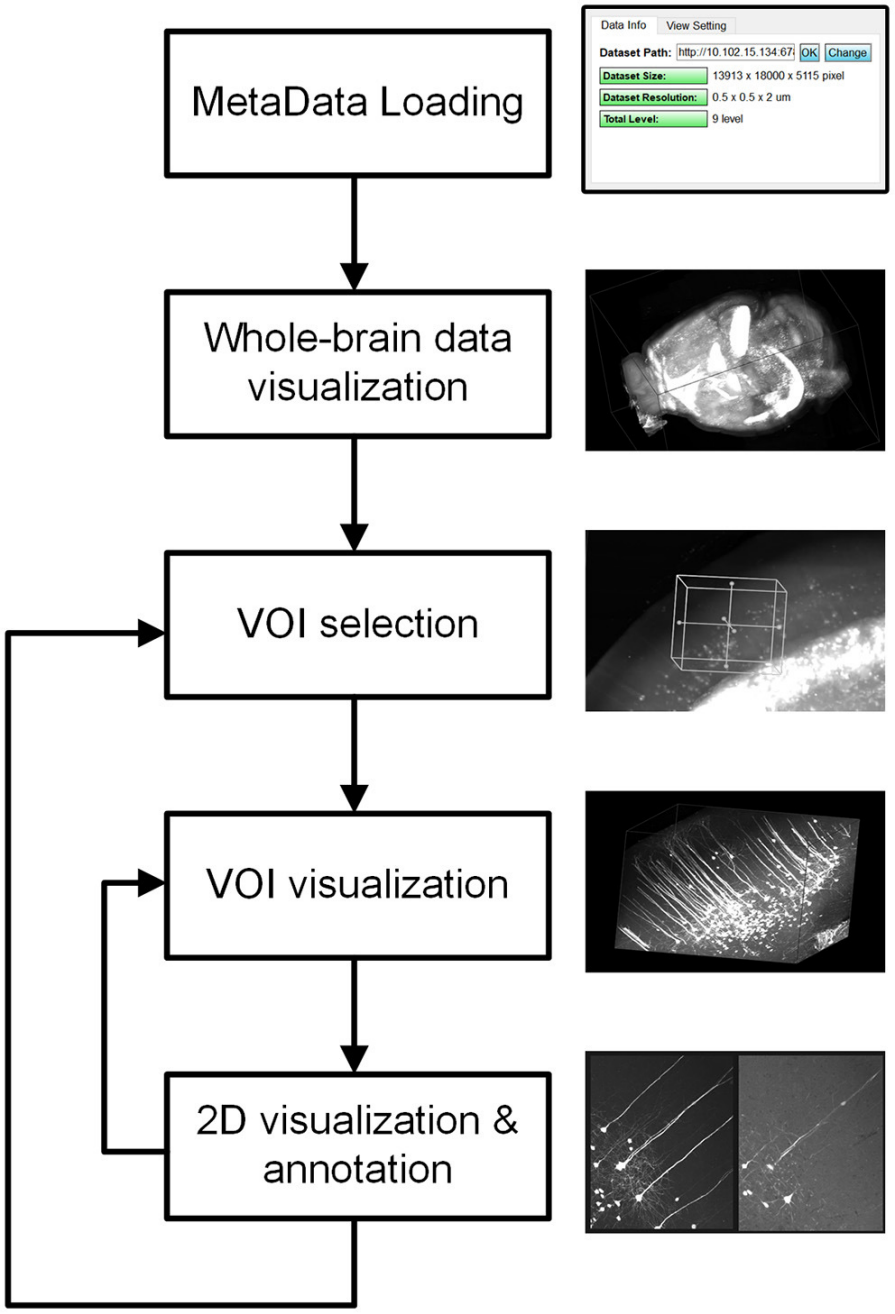
Interaction style workflow of webTDat. The entire dataset can be browsed by continuously selecting VOIs. The VOIs are set in 3D and annotated in 2D. VOIs and their annotated results can be visualized in 3D or 2D.

Restricted by the resolution and performance of typical computer displays and interactive devices, it is impossible to interact directly with terabyte-sized volume images. By analyzing the requirements of user interactions with mesoscopic-scale whole-brain datasets during tasks such as neuron tracing, brain registration, segmentation, and annotation, we devised a novel volume interaction style based on drag-click operations (Figure 4). Users can interactively select VOIs from a large-scale volume through simple computer-mouse actions (drag, scroll, and click operations). webTDat includes two main types of interactive functions: 3D interactive navigation and 2D interactive annotation.

The 3D interactive navigation procedures are as follows. First, the client loads metadata from the server side to determine the entire dataset's spatial coordinates. The client then loads the entire dataset at low resolution from the server and renders it to provide a 3D overview for users. A 3D rubber-band box is supplied for selection and navigation during data interaction in the interactive window. The region inside the navigation window is the VOI that needs to be loaded. The size and position of the 3D box can be changed using drag-and-drop operations. Finally, the client sends a request to the server to transmit the bitBlocks corresponding to the VOI. The VOI is gradually dynamically rendered from high-to-low bit-planes, as discussed above. The resolution level *R* of a VOI is determined by the size of the navigation window and calculated as follows:

(1) $$\begin{array}{l} R=\left[\log_{2}\frac{\left(x_{\max}-x_{\min}\right)\times \left(y_{\max}-y_{\min}\right)\times \left(z_{\max}-z_{\min}\right)}{1024\times 1024\times M}\right] \end{array}$$

where *x*_*min*_*, x*_*max*_*, y*_*min*_*, y*_*max*_*, z*_*min*_*, z*_*max*_ represent the maximum and minimum values in the x, y, and z directions of the navigation window, respectively. *M* represents the upper limit of the size of VOI that can be loaded each time (units: MB). The default setting is 20, ensuring that the process will not load more than 20 MBvoxel at a time.

Two D interactive annotation: After the VOI has been rendered on the client side, it can be displayed in a 2D model. webTDat provides two kinds of 2D visualization models: maximum intensity projection (MIP) mode and slice mode. The MIP mode projects the 3D VOI to 2D planes (X-Y, Y-Z, and X-Z planes). The users can control the thickness of the projection. The slice mode shows three orthogonal slices for the current VOI. The position of the projection and the slice can be switched by moving the mouse wheel. In the 2D interaction model, webTDat provides an interface for data annotation. Three types of vector structure data are used to save annotation results: point, line (tube), and 2D closed-contour surface. The annotation interface can be activated by clicking the mouse button. Users can add annotations either manually or automatically based on the webTDat annotation interface while webTDat completes the data I/O and rendering tasks.

### Implementation

The webTDat framework is mainly written in C++ and uses CMake for cross-platform deployment. Some open-source libraries are used; these mainly include Qt5 for the GUI, VTK for the rendering and interaction engine, libTIFF and OpenCV for image processing, and Nginx for the HTTP server. The software framework is publicly available on GitHub for noncommercial use.

### Source of Datasets and Computing Environment

Two mesoscopic-scale whole-brain imaging datasets were used in this work. Dataset1 is a Thy1-EGFP M-line transgenic mouse whose whole brain was imaged using a two-photon fluorescence Micro-Optical Sectioning Tomography system (2p-fMOST) (Zheng et al., 2013). The voxel resolution is 0.32 × 0.32 × 2 μm, the voxel size is 13,913 × 18,000 × 5115, and the raw data size is 1.17 TB. The original animal study was reviewed and approved by the Institutional Animal Ethics Committee of Huazhong University of Science and Technology, and detailed acquisition information can be found in the article. Dataset2 constitutes the complete cerebellum of an L7-GFP mouse acquired by confocal light sheet microscopy (CLSM). The voxel resolution is 0.8 × 0.8 × 1 μm, the voxel size is 3,662 × 8,249 × 3,646, and the raw data size is 102.6 GB. The raw data are publicly available from Harvard Dataverse (Silvestri, 2015a,b), and detailed information can be found in the article (Silvestri et al., 2015).

The computing configuration used in this study was a computer equipped with an i9-9900k CPU with 64 GB of RAM and a GTX1060 GPU with 6 GB of RAM as well as a 6 TB HDD. This computer was is used to generate the data format and provide client-side services. The server was configured with an i5-4570 CPU, 8 GB of RAM, an 8 TB HDD and a 100 Mbps Ethernet connection.

## Results

### Performance of the Bit-Plane Separated Data Format

**Figure 5:**
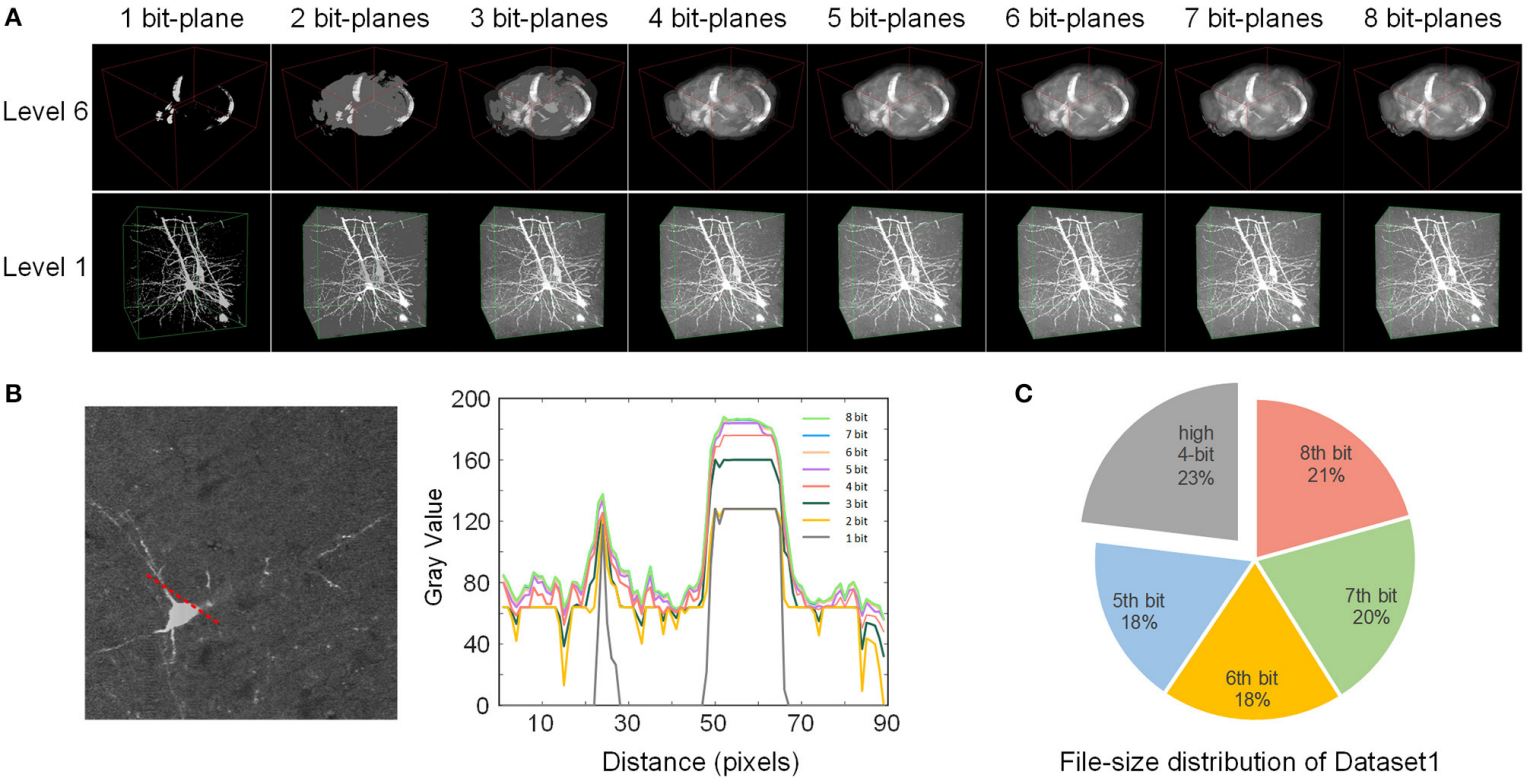
Effect of the bit-plane-separated data format. **(A)** The rendering quality of two VOIs at different bit depths. The depth of the displayed VOIs increases gradually from left to right. **(B)** The intensity profile of the 2D slice along the red dotted line at different bit depths. The slice was selected from the level 1 VOI above. A plot profile of Fiji acquired the numerical value. **(C)** The file size of each bit-plane of Dataset1. The first four bit planes are shown together.

We analyzed the effect of the bit-plane separated data format based on image rendering quality and the size of the data encoded by different bit-planes (Figure 5). The results showed that this format achieves an excellent performance with the described fine-grained data division, and it proved suitable for network transmission and visualization.

We converted Dataset1 into the webTDat format and then selected two VOIs with different resolution levels for visualization (Figure 5A). The time consumption required for conversion is shown in Table 1. The data at different bit-planes are read from high to low and combined to display the VOIs at different bit depths. When only one bit-plane (the highest bit-plane) of data was read and visualized, only the rough contours in the image were displayed. However, as the number of bit-planes of data read increased, the visualization effect became increasingly sharp, and the detail visible in the image became more abundant. After the data of 4 bit planes were read, as the number of visualized bit planes increased, the visualization effect changed little. We selected a 2D slice from the VOI in Figure 5A (level 1), drew a radial line on the slice image and plotted the gray values along this line at different bit depths (Figure 5B). When the bit depth of the image exceeded 4 bits, the gray values changed very little.

**Table 1:** Comparison of data reformatting performance between webTDat and TDat.

	**The number of levels**	**The number of files**	**Total file size**	**Time consumption**	**Memory consumption**
TDat	7	11,578	561 GB	7.86 h	3.5 GB
webTDat	9	5,635,504	718 GB	24.56 h	300 MB

In addition, we also counted the file size of each bit-plane after Dataset1 was converted into webTDat format (Figure 5C). The highest four bit-planes occupied less than 30% of the storage space but stored the dataset's primary information. In contrast, the lowest four bit-planes occupied more than 70% of the size and stored details of the dataset; however, they had little impact on the visualized data. Therefore, users can reduce the rendering bit depths according to the desired data quality to reduce the amount of data required for visualization.

We note that the image quality of Dataset1 is good, with high image contrast. Therefore, the data in the lowest four bit-planes have little influence on the image's visualization effect. If contrast, when an image has low contrast, the information stored in the low four bit-planes of the image will significantly affect the image quality. Some comprehensive image processing algorithms could be applied to improve the contrast of the dataset during image preprocessing. The main point here is to show that webTDat separates images into bit-planes, forming the basis of the visualization approach described above.

### Benchmarks for Data Reformatting

We recorded various performance parameters when converting Dataset1 to webTDat format and compared these parameters with similar results from TDat (Table 1). Compared with TDat, webTDat decreases the block size from 512^3^ voxels to 128^3^ voxels, and split each block into bitBlocks for storage. As a result, webTDat supports more resolution levels and comprises more files (three orders of magnitude higher) than does TDat. Because of the large number of files, the lossless compression efficiency of webTDat was slightly lower, and its conversion takes longer than that of TDat. However, webTDat offers reduced memory consumption during reformatting because the block size is reduced compared with TDat. Although webTDat is slower than TDat, it can process ~1 terabyte of data per day on a common computer. Moreover, webTDat's reformatting performance is still highly efficient compared to other similar software (Li et al., 2017). webTDat adopts the same FPR algorithm as TDat for data reformatting. This algorithm could be extended to more efficient computing platforms to achieve higher performance, such as workstations and computer clusters.

### The Performance of Dynamic Rendering

**Figure 6:**
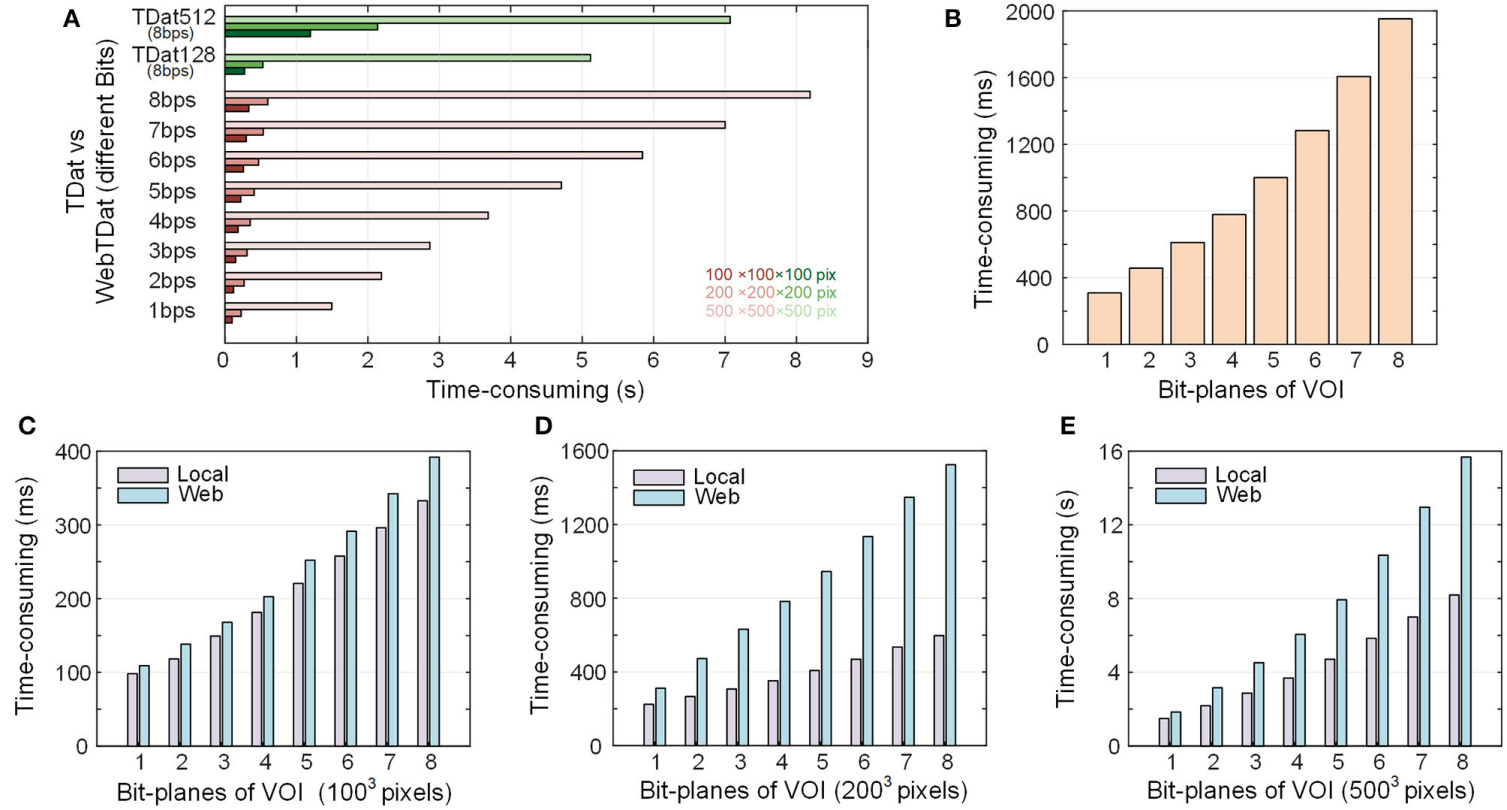
Time consumption to render VOIs from the webTDat dataset: **(A)** Time consumption to render VOIs at three fixed sizes with webTDat compared to the time consumption required by the original TDat (TDat512) and TDat128. The webTDat results are displayed according to different bit depths. The different colors represent different ROI sizes. This benchmark was conducted in a stand-alone environment. **(B)** Time consumption to render VOIs of random size by webTDat. The results are displayed according to different bit depths. **(C–E)** Comparison of VOI rendering times between local and networked environments. The VOI sizes are 100^3^, 200^3^, and 500^3^ pixels. All the benchmarks were repeated more than 20 times for each ROI size, and the average runtime was calculated. The numerical data can be found in the Supplementary Table.

We tested the performance of data loading during visualization. The results showed that the dynamic image rendering method adopted by webTDat effectively reduces the response time during the visualization process (Figure 6). We visualized three different VOIs randomly selected from Dataset1, which was converted to webTDat format. The sizes of the VOIs are 100^3^, 200^3^, and 500^3^ voxels, respectively. We counted the time consumption of loading these VOIs during visualization (Figure 6A).

In contrast, we converted the same dataset into TDat format and counted the time consumption required to load the same VOI with TDat. Since the original TDat block size (512^3^ voxels) is inconsistent with webTDat (128^3^ voxels), we also set the TDat block size to 128^3^ pixels (called TDat128) and then compared it with webTDat. Because TDat does not read data through the network, the dataset was stored locally during the test. webTDat has a short response time when loading data. For the 100^3^- and 200^3^-voxel VOI sizes, webTDat consumed less than 200 ms to load the highest bit-plane data and render the results. In contrast, TDat require reading all data at full-bit depth before rendering the VOIs; thus, its response time exceeded 1 s. Because the sizes of webTDat blocks are smaller than those of TDat, the required data can be loaded at a finer granularity when rendering VOIs, which reduces the amount of redundant data loaded while reading. Interestingly, even when webTDat loaded all the bit planes (eight bits) of data, its time consumption was still less than that of TDat. For the 500^3^-voxel size VOI, webTDat required only 1.5 s to load the highest bit-plane data, while TDat consumed more than 7 s to load the data. Although TDat128 and webTDat have the same block granularity, the load speed of webTDat is faster than that of the original TDat. Even TDat128 must still load all the bit planes when rendering VOIs; consequently, the response time of webTDat was still significantly lower than that of TDat128 (Figure 6A). While TDat128 loaded all 8 bit-planes data, webTDat could loaded 6-7 bit-planes data, which was sufficient to display most of the information.

We also tested the time consumption of webTDat when loading data through the network compared to loading data through the local network (Figures 6C–E). The sizes of VOIs were the same 100^3^, 200^3^, and 500^3^ voxels. Loading the data through the network took slightly more time than loading through the local network. Simultaneously, the sizes of the high bit-plane files were small, resulting in less network transmission time consumption; therefore, the response times did not change substantially. However, the sizes of the low bit-plane files are larger; thus, with more bit-plane data to load, the time consumption increased significantly.

In the tests described above, we tested the time consumption during visualization by loading fixed-size VOIs. However, in practice, webTDat does not load large-scale data during visualization; instead, it calculates the resolution level at which to load data automatically based on the size of the interaction window, which determines the voxel sizes of the VOIs. We used the interaction window to randomly select VOIs with different sizes at diverse locations to simulate realistic usage conditions and counted the time consumption needed to visualize these VOIs. The results showed that the data response time during visualization was less than 400 ms and that the time to load all bit-planes of data was <2 s (Figure 6B).

### Interactive Visualization for Large-Scale Volumes

**Figure 7:**
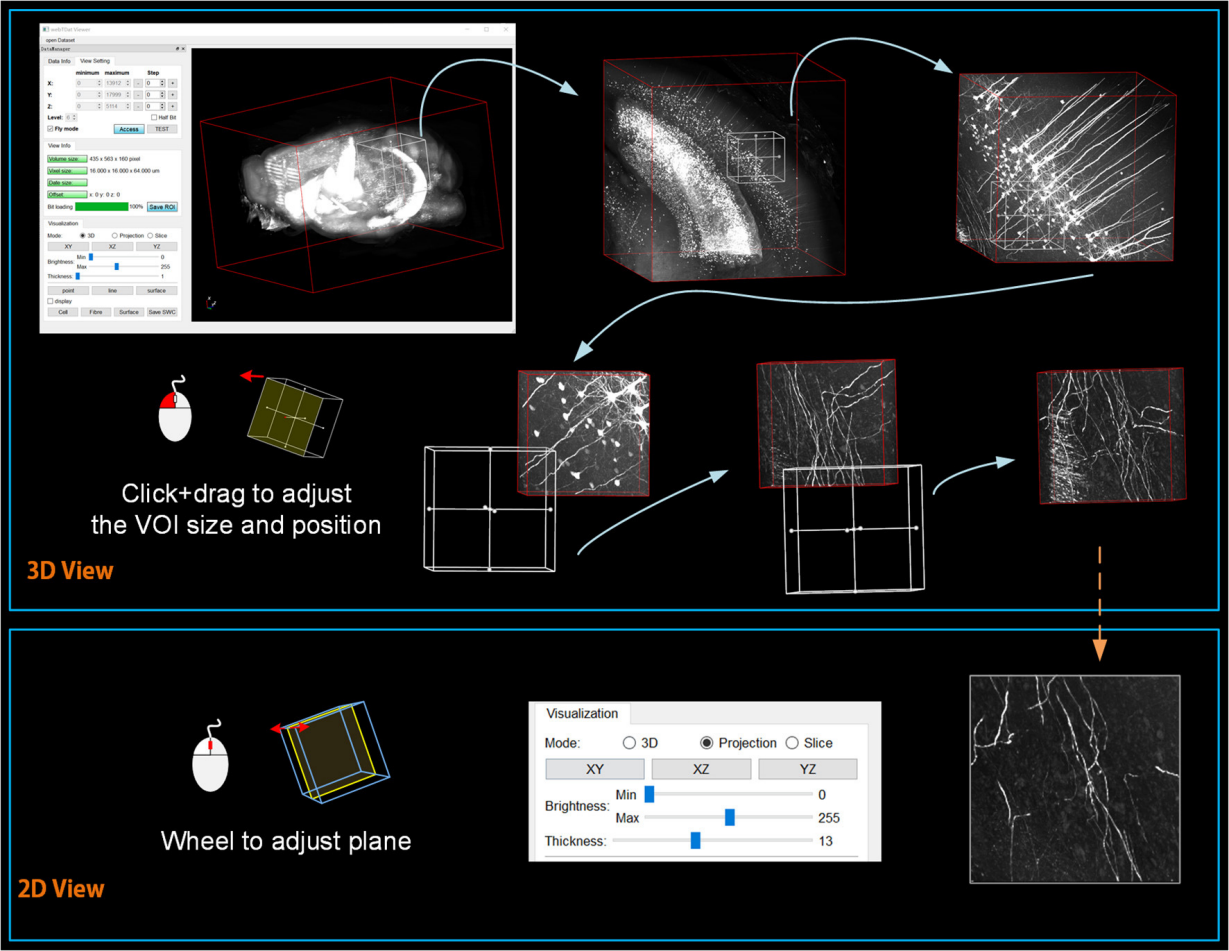
The webTDat interface when interactively browsing whole-brain data. The interactive window can be slid throughout the entire data space to select VOIs, which can be displayed in 3D or 2D modes.

webTDat allows users to browse mesoscopic whole-brain data interactively with simple computer-mouse actions. We used webTDat to perform interactive visualizations of Dataset1 (Figure 7). First, the rough (low resolution) whole-brain data gives users an outline. Then, by sliding the interactive window, the VOI scope is gradually narrowed, and the resolutions of the loaded VOIs improved. The interactive window can be used to select VOIs from the entire data space or to slide along a structure of interest in the volume. Users could browse the VOIs by switching modes, which include the 3D, MIP, and slice modes (see the Supplementary Movie).

### Support for Remote Data Annotation and LSM Data

**Figure 8:**
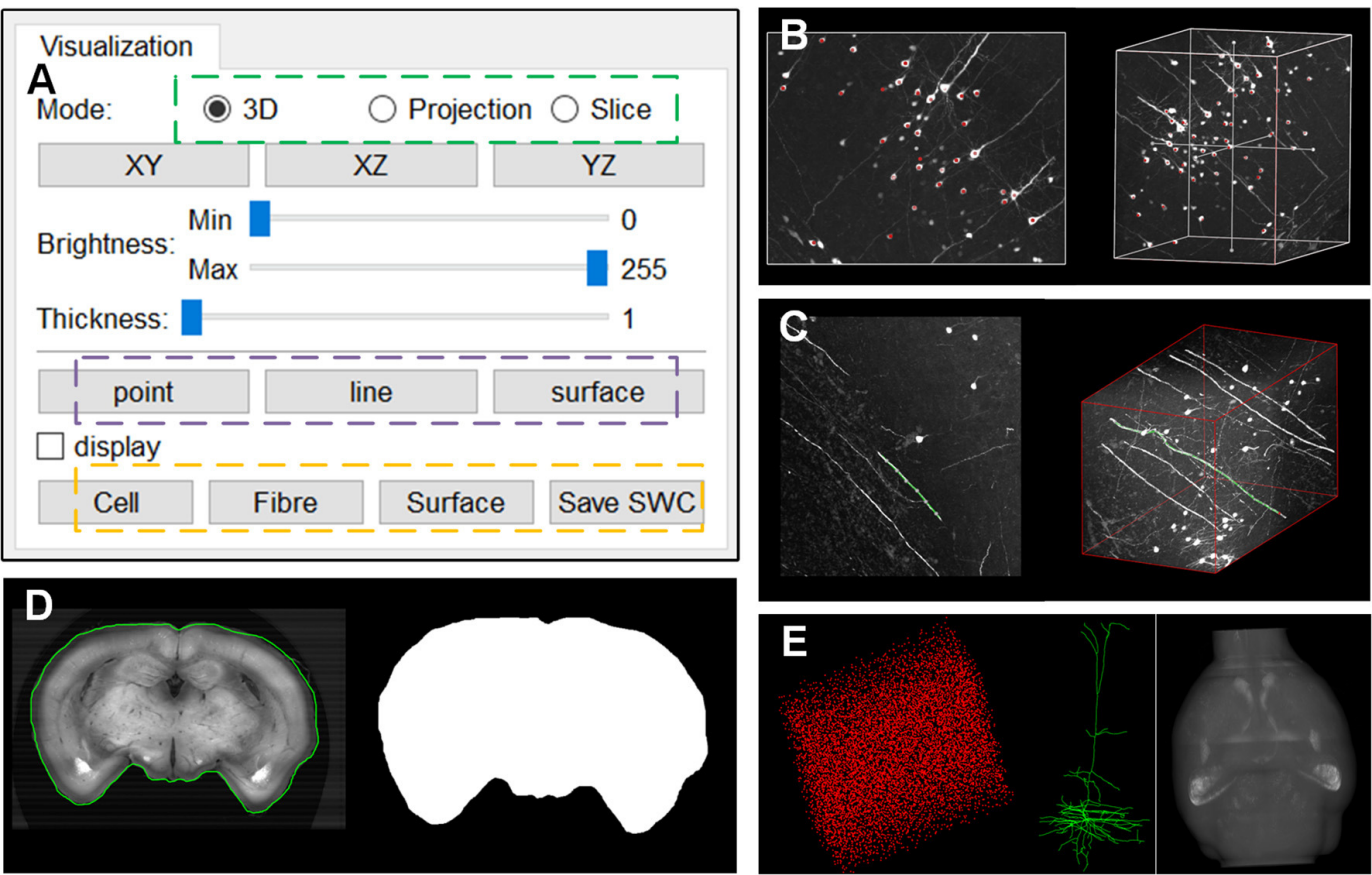
The webTDat interface for data annotation: **(A)** The control panel for activating the annotation functions; **(B)** Annotation of somas in “point” mode in the MIP view; **(C)** Annotation of neuron fibers in “line” mode in the MIP view; **(D)** Annotation of brain contour in “surface” mode in the slice view; **(E)** Visualizations of the three types of annotation results, namely, soma, neuron, and brain contour.

webTDat provides interactive data visualization capabilities and offers rich interfaces for data annotation, which are integrated into the webTDat viewer. We demonstrated the effect of data annotation on Dataset1 (Figure 8). The annotation function is activated by buttons in the visualization panel (Figure 8A). Three types of annotation modes are provided, corresponding to “point,” “line,” and “surface.” The first two modes must be activated in MIP mode, while the “surface” mode can be activated in slice mode. Figure 8B shows some sample annotation results of the cell body (somas) of neurons in VOI; these somas were stored as “point” structures. Figure 8C shows the annotation results of neuron fibers, where the annotation results were saved as “line” structures. Figure 8D shows how “surface” structures can be used to store annotation results for the brain contour in slice mode. A binary mask of the current slice could be generated automatically by outlining the brain contour. webTDat also provides various I/O capabilities for the annotation results, which can be read, written, and visualized in webTDat viewer (Figure 8E).

**Figure 9:**
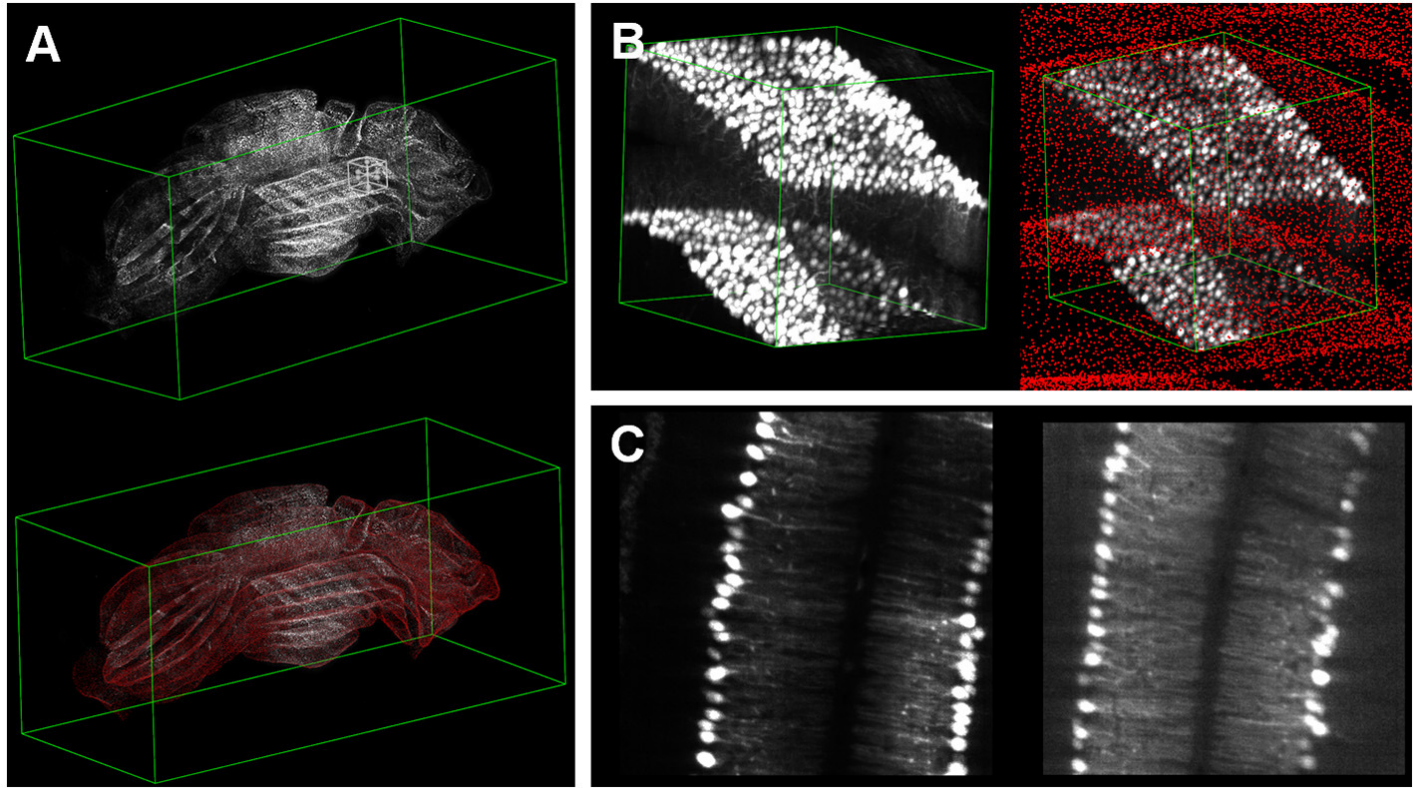
Visualization of whole mouse cerebellum: **(A)** 3D visualization of the whole dataset with low resolution and Purkinje cells; **(B)** 3D visualization of a VOI selected from the interactive window in **(A)**; **(C)** MIP and slice modes displaying the VOI.

webTDat can also be used to visualize datasets generated by other imaging systems, especially those generated by whole-brain optical microscopy. Figure 8 shows a visualization of mouse cerebellum data generated by CLSM (Dataset2). The raw data were reformatted into webTDat format and stored on the server; then, the data could be browsed in a variety of ways through the webTDat viewer from any location (Figure 9). The webTDat framework can be used to visualize any 3D biological image, and its advanced rendering and visualization methods can be applied to almost any type of image.

## Discussion

This study proposed webTDat, a web-based, real-time 3D interactive visualization framework for large-scale volume images. Although webTDat still uses the octree and the C/S architecture, this study makes innovations based on those two methods to remove the bottleneck between the need to transmit large-scale data and the limited bandwidth available for network-based visualization. We designed a bit-plane-separated, fine-grained storage structure to separate images by pixel bit depth based on the octree. In this way, the data can be read with finer granularity during visualization, and the amount of data required for visualization is reduced. Based on the C/S architecture, a dynamic image rendering scheme is adopted to allow users to interact with the data while it is being transmitted from the server to the client. This approach reduces the time required for data loading during visualization and responds first with the data most needed for interaction. The webTDat client provides rich visualization and interaction functions for data browsing as well as interfaces for data annotation. The webTDat system can easily be expanded and used to process massive data generated by various imaging systems.

The bit-separated storage format used by webTDat can be used not only for dynamic data rendering for visualizations but also for image registration and image segmentation purposes. For example, the primary information stored in a high bit-plane of the image could be processed first, and then the low bit-planes of the image could be processed gradually. Alternatively, the low-resolution data could be processed first, and the high-resolution data could be processed iteratively. This approach allows the same data format to satisfy the needs of different image processing tasks and improve data utilization efficiency.

Although webTDat has tremendous advantages for large-scale volume visualizations, it still has some shortcomings that need to be noted. On one hand, the final bitBlock files cannot be stored with lossy compression because bitBlocks store different bit planes of pixels in separate files. The same bit-plane in eight pixels forms a byte in one bitBlock file; therefore, the value of each byte in the bitBlock is not directly related to the gray value of a pixel. Consequently, using lossy compression on bitBlocks can change the byte values, which will affect some bits in the pixels. If these bits are the image's high bit planes, the image's gray value can change substantially. Therefore, the final encoded files must be stored using lossless compression, which limits the compression rate of the final encoded file. However, webTDat adopted the C/S architecture, which requires keeping only one copy of the dataset. In addition, before the original data are converted to webTDat format, the data can be preprocessed and denoised to smooth the gray value changes between adjacent pixels. This operation can reduce the size of the final encoded file. On the other hand, webTDat uses a small block size, and each block is stored in multiple files. This splits one dataset into an enormous number of mostly small files. If the dataset is extremely large, the resulting large number of small files can place considerable pressure on the server's file system. When designing the webTDat format, we investigated the best block size and compared it with many similar tools to determine the proper size. In practice, we found that terabyte-scale data does not suffer from this problem. However, if the data are more massive, the problem could be improved using more efficient file indexing (e.g., a database system) or by improving the system's hardware performance (e.g., using an SSD).

Based on TDat, webTDat achieves the ability to visualize large-scale volume data. webTDat inherits many of the advantages of TDat and further improves TDat's functionality. webTDat viewers could be altered to support the visualization of TDat datasets with only a few modifications. We believe that webTDat can be as powerful a tool as TDat for neuroscience researchers.

## Data Availability Statement

The datasets generated for this study are available on request to the corresponding author. The code is available on GitHub (https://github.com/visionlyx/webTDat).

## Ethics Statement

The animal study was reviewed and approved by the Institutional Animal Ethics Committee of Huazhong University of Science and Technology.

## Author Contributions

YL designed the method. YL, JL, and TC developed the software. YL and AL wrote the article. AL provided the datasets. HZ, HW, and KW tested software. All authors contributed to the article and approved the submitted version.

## Conflict of Interest

The authors declare that the research was conducted in the absence of any commercial or financial relationships that could be construed as a potential conflict of interest.
